# Baseline Patient and Tumour Characteristics Associated with Mental Health in Men Undergoing Active Surveillance for Prostate Cancer

**DOI:** 10.1002/bco2.70293

**Published:** 2026-10-15

**Authors:** Beth Russell, Mia Tazi, Harriet Wylde, Alan Francis, Christian P. Pavlovich, Michael Peacock, Daan Nieboer, Lionne D. F. Venderbos, Prokar Dasgupta, Mieke Van Hemelrijck, Global Active surveillance data research Platform (GAP3) consortium

**Affiliations:** ^1^ Transforming Cancer Outcomes Through Research (TOUR) King's College London London UK; ^2^ Johns Hopkins School of Medicine The James Buchanan Brady Urological Institute Baltimore Maryland USA; ^3^ University of British Columbia, BC Cancer Agency Vancouver Canada; ^4^ Department of Urology, Erasmus MC Cancer Institute Erasmus University Medical Center Rotterdam The Netherlands; ^5^ Department of Urology Guy's Hospital Guy's and St Thomas' NHS Foundation Trust London UK

**Keywords:** active surveillance, mental health, mental wellbeing, prostate cancer

## Abstract

**Objectives:**

This study aims to examine how patient demographics and clinical characteristics relate to mental well‐being in men with prostate cancer (PCa) on active surveillance (AS).

**Subjects and Methods:**

We analysed data from the GAP3 database, established in 2014. The study included men from three cohorts who completed baseline self‐reported well‐being assessments using either the Memorial Anxiety Scale for Prostate Cancer (MAX‐PC, *n* = 488) or the Functional Assessment of Cancer Therapy–Prostate Emotional Well‐being Score (FACT‐P EWB, *n* = 497). MAX‐PC scores were categorised as mild (<9), moderate (9–27) or high anxiety (≥27), while FACT‐P EWB scores were divided into tertiles (≤18, 19–20 and ≥21; higher scores indicate better emotional well‐being). Descriptive statistics and ordinal logistic regression were used to examine associations between baseline characteristics and questionnaire scores.

**Results:**

Among the men across three cohorts, 488 completed the MAX‐PC and 497 the FACT‐P EWB. In the chi2 analysis for the MAX‐PC cohort, anxiety levels were significantly associated with baseline BMI, ethnicity, marital status, education, family history of PCa, PSA levels, T stage, smoking status, Charlson Comorbidity Index and erectile dysfunction (all *p* < 0.001). Ordinal logistic regression showed that a family history of prostate cancer was associated with higher anxiety (OR = 1.94, 95% CI: 1.26–3.00). In contrast, no significant associations were observed in the FACT‐P EWB cohort.

**Conclusion:**

Our findings highlight several baseline demographic and clinical factors associated with disease‐specific anxiety in men with PCa on AS. These results underscore the importance of routine psychosocial elements and of considering lifestyle and psychosocial factors when evaluating the supportive care needs of men considering or pursuing PCa AS.

## 1 INTRODUCTION

Prostate cancer (PCa) is the most common cancer among men in Europe and second most common in the world.^1^, ^2^, ^3^ While advancements in diagnostic and therapeutic strategies have significantly improved survival rates, they have also introduced complex challenges in the management of low‐risk PCa. Active surveillance (AS), a strategy involving regular monitoring of the cancer's progression without immediate intervention, has been established as a preferred approach for men with low‐risk PCa.^4^ Follow‐up methods include ongoing prostate specific antigen (PSA) checks, MRIs, digital rectal examinations (DREs) and prostate biopsies. This approach aims to reduce overtreatment and avoid the side effects associated with radical therapies, such as surgery or radiotherapy. However, while AS minimises adverse events from treatments, its psychological impact, particularly on mental health, is an area of interest.

With cancer patients living longer than ever,^5^ there is increased emphasis on not just quantity but also quality of life for cancer patients. Mental wellbeing is just one factor impacting a patient's quality of life and addressing this has become an integral component of holistic cancer care. National guidelines, such as those from the National Institute for Health and Care Excellence (NICE), emphasise the importance of incorporating psychosocial support into PCa management; however, these guidelines mainly surround the adverse effects of treatment rather than surveillance.^6^

Men who are on AS may experience heightened anxiety, depression or stress as a result of their untreated cancer with fears and uncertainty around prognosis and progression as well as regarding ongoing testing. Results from previous studies have been varied in terms of looking at the overall mental wellbeing of patients on AS.^7^, ^8^, ^9^ However, there is a paucity of studies investigating the interplay between baseline clinical and demographic characteristics and mental wellbeing for men on AS. Therefore, the present study aims to identify baseline demographic and clinical characteristics that may be associated with mental wellbeing in men on AS using data from questionnaires.

## 2 METHODS

### 2.1 Data source

The Global Action Plan Prostate Cancer Active Surveillance (GAP3) is the largest global PCa AS database.^10^ The GAP3 cohort was set out to create a global consensus on the selection and monitoring of men with low‐risk PCa. The GAP3 initiative currently consists of 24 cohorts with nearly 27,000 from 14 countries spanning Europe, the United Kingdom, the United States, Canada, Asia and Australia. A requirement for participation in GAP3 is that each contributing institution must have the relevant ethical approvals for contributing anonymised patient‐level data to the GAP3 database.

A wealth of data points is included within the GAP3 database including baseline demographic and clinical information, as well as follow‐up information on factors such as PSA, PSA kinetics, T stage by DRE, biopsy characteristics and MRI findings. Further to these, data on the discontinuation of AS, subsequent treatments and cause of death are also collected. Some centres also contributed data obtained from questionnaires, which serve the focus of this study.

### 2.2 Study cohort

Using the GAP3 database (V5 release date May 2025), patients with low‐risk PCa, who completed self‐reported wellbeing questionnaires at baseline (within 90 days of starting AS), were selected for inclusion into the study. The data included in the study were from three cohorts (MD Anderson Cancer Centre, Milan and Erasmus MC). Two cohorts of patients were subsequently created based on completed questionnaires: the Memorial Anxiety Scale for Prostate Cancer (MAX‐PC) and the Functional Assessment of Cancer Therapy–Prostate Emotional Wellbeing Score (FACT‐P EWB). Patients who did not submit the relevant wellbeing questionnaires were excluded from the study. The MAX‐PC cohort included data from three cohorts (MD Anderson Cancer Centre, Fondazione IRCCS Istituto Nazionale dei Tumori di Milano and Erasmus MC) while the FACT‐P EWB cohort included data from Milan only.

### 2.3 Sociodemographic and clinical variables

The following baseline variables were obtained: Age (<50, 50–59, 60–69, 70–90, 80+), BMI (healthy: 18.5–≤25, overweight 25–≤30, obese >30), ethnicity (Hispanic or Latina, Non‐Hispanic), marital status (married, not married), highest Level of education (less than high school, high school, university), family history of PCa (yes/no), PSA (<1, 1–4, 5–9, 10+), T‐Stage (T1, T2), number of biopsy cores with cancer (1–4, 5–9), Gleason Score (<7, 7), smoking history (never, former, current), Charlson Comorbidity Index (CCI) (0, 1, 2, 3+) and erectile dysfunction (yes/no).

### 2.4 Wellbeing measures

MAX‐PC comprises a 24‐item scale that has been created to assess three components of PCa‐related anxiety: general anxiety about PCa and treatment, fear of recurrence and anxiety surrounding PSA testing.^11^ To make results comparable, the patients' scores were categorised using previously defined categories^12^: mild (MAX‐PC < 9), moderate (MAX‐PC 9–27) and high anxiety (MAX‐PC ≥ 27).

The FACT‐P evaluates Health related Quality of Life (HRQoL) in males with PCa.^13^ FACT‐P is made up of FACT‐G (general), a 27‐item self‐report questionnaire that assesses general HRQoL in cancer patients, and a 12‐item PCa subscale (PCS). The PCS is particularly intended to assess PCa‐related quality of life. The emotional wellbeing (EWB) section of the FACT‐P questionnaire focusses on addressing the patient's morale, perception of hope and nervousness, with regard to their PCa. Six questions were asked, scored from 0 (*not at all*) to 4 (*very much*). The total score for this section ranges from 0 to 24. As there are no pre‐defined categories for FACT‐P EWB, tertiles were used for analysis, with a higher score indicating a better quality of life (QoL).

### 2.5 Statistical analysis

The cohorts were first described using descriptive statistics including Chi‐squared tests. Ordinal logistic regression analyses were then applied to assess the association between the sociodemographic and tumour characteristics and MAX‐PC or FACT‐P EWB scores. Directed acyclic graphs (DAGs) were used to determine the minimal adjustments required when considering each exposure and outcome association one at a time. All analyses were conducted using StataMP version 18 (Texas, USA).

## 3 RESULTS

The present study involved 985 men, from three centres who completed self‐reported data on well‐being (either via the MAX‐ PC [*n* = 488] or the FACT‐P EWB [*n* = 497]). In the MAX‐PC cohort, 26% of patients reported mild anxiety (MAX‐PC < 9), 46% moderate anxiety (MAX‐PC 9–27) and 28% reported severe anxiety (MAX‐PC > 27). For the FACT‐P EWB score, 37% were in the lowest tertile indicating a lower EWB (score of ≤18), 27% in the middle tertile (score of 19–20) and 36% were in the highest tertile (score of ≥21) indicating a higher EWB.

In the MAX‐PC cohort, men were diagnosed between 2007 and 2017. At baseline, most men were aged over 60 (71%) (mean age = 63), non‐Hispanic (90%), married (60%) and reported no family history of PCa (69%) (Table 1). In the chi‐squared analyses, BMI was associated with anxiety (*p* < 0.001); patients who were obese were more likely to have high levels of anxiety as compared to those who were non‐obese. Ethnicity and marital status also showed significant variation (both *p* < 0.001), with non‐Hispanic and married men more likely to report lower anxiety. Education level was inversely related to anxiety (*p* < 0.001), with lower education more common in the severe group. A family history of PCa was more prevalent among those with severe anxiety (*p* < 0.001). PSA levels, T stage, smoking status, Charlson Comorbidity Index (CCI) and erectile dysfunction were also significantly associated with anxiety levels (all *p* < 0.001).

**TABLE 1:** Cohort characteristics of MAX‐PC cohort.

Category	Total		Mild		Moderate		High		Chi2
	*n*	%	*n*	%	*n*	%	*n*	%	
	*n* = 488		*n* = 127		*n* = 224		*n* = 137		
Age at diagnosis									
<50	13	2.7	3	2.40	4	1.80	6	4.40	0.110
50–59	129	26.4	27	21.30	60	26.80	42	30.70	
60–69	235	48.2	60	47.20	118	52.70	57	41.60	
70–79	110	22.5	37	29.10	42	18.80	31	22.60	
≥80	1	0.2	0	0.00	0	0.00	1	0.70	
BMI									
Healthy: 18.5–≤25	149	30.5	35	27.60	90	40.20	24	17.50	<0.001
Overweight: 25–≤30	195	40	54	42.50	94	42.00	47	34.30	
Obese: >30	68	13.9	18	14.20	11	4.90	39	28.50	
Missing	76	15.6	20	15.70	29	12.90	27	19.70	
Ethnicity									
Hispanic or Latino	5	1	0	0.00	1	0.40	4	2.90	<0.001
Non‐Hispanic	435	89.1	120	94.50	211	94.20	104	75.90	
Missing	48	9.8	7	5.50	12	5.40	29	21.20	
Marital status									
No	44	9	17	13.40	20	8.90	7	5.10	<0.001
Yes	292	59.8	99	78.00	172	76.80	21	15.30	
Missing	152	31.1	11	8.70	32	14.30	109	79.60	
Education									
High school	86	17.6	37	29.10	38	17.00	11	8.00	<0.001
Less than high school	120	24.6	37	29.10	76	33.90	7	5.10	
University	75	15.4	27	21.30	42	18.80	6	4.40	
Missing	207	42.4	26	20.50	68	30.40	113	82.50	
Family history of PCa									
No	336	68.9	99	78.00	169	75.40	68	49.60	<0.001
Yes	102	20.9	21	16.50	41	18.30	40	29.20	
Missing	50	10.2	7	5.50	14	6.20	29	21.20	
PSA									
<1	11	2.3	1	0.80	3	1.30	7	5.10	<0.001
1—4	218	44.7	49	38.60	86	38.40	83	60.60	
5—9	258	52.9	76	59.80	135	60.30	47	34.30	
10+	1	0.2	1	0.80	0	0.00	0	0.00	
T stage									
T1	440	90.2	114	89.80	213	95.10	113	82.50	<0.001
T2	48	9.8	13	10.20	11	4.90	24	17.50	
No. of biopsy cores with cancer									
1–4	480	98.4	125	98.40	220	98.20	135	98.50	0.862
5–9	6	1.2	1	0.80	3	1.30	2	1.50	
Missing	2	0.4	1	0.80	1	0.40	0	0.00	
Gleason									
<1	485	99.4	127	100.00	223	99.60	135	98.50	0.117
7	2	0.4	0	0.00	0	0.00	2	1.50	
Missing	1	0.2	0	0.00	1	0.40	0	0.00	
Smoking									
Never	131	26.8	40	31.50	82	36.60	9	6.60	<0.001
Former	84	17.2	30	23.60	49	21.90	5	3.60	
Current	98	20.1	36	28.30	49	21.90	13	9.50	
Missing	175	35.9	21	16.50	44	19.60	110	80.30	
CCI									
0	50	10.2	9	7.10	13	5.80	28	20.40	<0.001
1	65	13.3	17	13.40	43	19.20	5	3.60	
2	125	25.6	43	33.90	72	32.10	10	7.30	
3+	78	16	34	26.80	35	15.60	9	6.60	
Missing	170	34.8	24	18.90	61	27.20	85	62.00	
Erectile dysfunction									
No	237	48.6	83	65.40	134	59.80	20	14.60	<0.001
Yes	45	9.2	18	14.20	26	11.60	1	0.70	
Missing	206	42.2	26	20.50	64	28.60	116	84.70	

In the FACT‐P EWB cohort, men were diagnosed between 2007 and 2018. In this cohort, at baseline, most men were aged over 60 (76%) (mean age = 64), non‐Hispanic (100%), married (66%) and had no family history of PCa (79%) (Table 2). None of the chi‐squared analyses were statistically significant for this cohort.

**TABLE 2:** Cohort characteristics of FACT‐P cohort.

Category	Total		FACTP ≤18		FACTP 19–20		FACTP ≥21		Chi2
	*n*	%	*n*	%	*n*	%	*n*	%	
	*n* = 497		*n* = 186		*n* = 133		*n* = 178		
Age at diagnosis									
<50	7	1.4	5	2.7	1	0.8	1	0.6	0.187
50–59	112	22.5	50	26.9	31	23.3	31	17.4	
60–69	245	49.3	85	45.7	65	48.9	95	53.4	
70–79	133	26.8	46	24.7	36	27.1	51	28.7	
BMI									
≤18.5	1	0.2	0	0	1	0.8	0	0	0.132
18.5–≤25	161	32.4	59	31.7	53	39.8	49	27.5	
25–≤30	187	37.6	75	40.3	42	31.6	70	39.3	
>30	42	8.5	14	7.5	7	5.3	21	11.8	
Missing	106	21.3	38	20.4	30	22.6	38	21.3	
Ethnicity									
Non‐Hispanic	497	100	186	100	133	100	178	100	N/A
Marital status									
No	51	10.3	16	8.6	15	11.3	20	11.2	0.559
Yes	329	66.2	122	65.6	84	63.2	123	69.1	
Missing	117	23.5	48	25.8	34	25.6	35	19.7	
Education									
Less than high school	124	24.9	44	23.7	32	24.1	48	27	0.624
High school	176	35.4	74	39.8	47	35.3	55	30.9	
University	111	22.3	35	18.8	33	24.8	43	24.2	
Missing	86	17.3	33	17.7	21	15.8	32	18	
Family history of PCa									
No	393	79.1	145	78	104	78.2	144	80.9	0.702
Yes	80	16.1	32	17.2	20	15	28	15.7	
Missing	24	4.8	9	4.8	9	6.8	6	3.4	
PSA									
<1	9	1.8	2	1.1	3	2.3	4	2.2	0.090
1–4	191	38.4	72	38.7	49	36.8	70	39.3	
5–9	287	57.7	111	59.7	74	55.6	102	57.3	
10+	10	2	1	0.5	7	5.3	2	1.1	
T stage									
T1	461	92.8	170	91.4	126	94.7	165	92.7	0.525
T2	36	7.2	16	8.6	7	5.3	13	7.3	
No. of biopsy cores with cancer									
1–4	480	96.6	179	96.2	130	97.7	171	96.1	0.506
5–9	12	2.4	6	3.2	1	0.8	5	2.8	
≥10	1	0.2	0	0	1	0.8	0	0	
Missing	4	0.8	1	0.5	1	0.8	2	1.1	
Gleason									
<7	496	99.8	186	100	132	99.2	178	100	0.254
7	1	0.2	0	0	1	0.8	0	0	
Smoking									
Current	186	37.4	68	36.6	54	40.6	64	36	0.638
Former	96	19.3	42	22.6	22	16.5	32	18	
Never	103	20.7	36	19.4	24	18	43	24.2	
Missing	112	22.5	40	21.5	33	24.8	39	21.9	
CCI									
0	4	0.8	3	1.6	0	0	1	0.6	0.295
1	70	14.1	29	15.6	24	18	17	9.6	
2	145	29.2	50	26.9	36	27.1	59	33.1	
3+	101	20.3	36	19.4	30	22.6	35	19.7	
Missing	177	35.6	68	36.6	43	32.3	66	37.1	
Erectile dysfunction									
No	334	67.2	117	62.9	93	69.9	124	69.7	0.506
Yes	47	9.5	19	10.2	10	7.5	18	10.1	
Missing	116	23.3	50	26.9	30	22.6	36	20.2	

The ordinal logistic regression analyses showed that men who had a family history of PCa (OR = 1.94, 95% CI 1.26–3.00) were at increased odds of having a higher MAX‐PC score (Table 3). Although obesity (BMI > 30) was not significantly associated with anxiety in unadjusted analyses, it became statistically significant after multivariable adjustment; however, this estimate was accompanied by a wide 95% confidence interval, indicating limited precision.

**TABLE 3:** Ordinal logistic regression results for MAX‐PC cohort.

Variable	Unadjusted OR	95% CI	Adjusted OR	95% CI	
Age at diagnosis					
<50	1.00	Ref.	1.00	Ref.	
50–59	0.7	(0.23–2.14)	0.6	(0.01–25.77)	
60–69	0.51	(0.17–1.51)	0.65	(0.01–51.67)	
70–79	0.45	(0.15–1.39)	0.22	(0.00–20.31)	
≥80	N/A				
BMI					
18.5–≤25	1.00	Ref.	1.00	Ref.	
25–≤30	1.09	(0.74–1.61)	0.88	(0.51–1.50)	
>30	3.26	(1.82–5.86)	0.28	(0.09–0.82)	
Marital status					
No	1.00	Ref.			*Unadjusted only
Yes	0.94	(0.49–1.81)			
Education					
Less than high school	0.77	(0.44–1.33)	0.77	(0.44–1.33)	
High school	1.00	Ref.	1.00	Ref.	
University	0.88	(0.50–1.55)	0.88	(0.50–1.55)	
Family history of PCa					
No	1.00	Ref.	1.00	Ref.	
Yes	2.13	(1.38–3.27)	1.94	(1.26–3.00)	
PSA					
<1	1.00	Ref.	1.00	Ref.	
1–4	0.32	(0.09–1.12)	1.46	(0.16–13.48)	
5–9	0.16	(0.05–0.55)	0.8	(0.09–7.33)	
10+	N/A				
T stage					
T1	1.00	Ref.	1.00	Ref.	
T2	1.99	(1.09–3.64)	1.01	(0.32–3.19)	
No. of biopsy cores with cancer					
1–4	1.00	Ref.	1.00	Ref.	*Unadjusted only
5–9	1.43	(0.33–6.26)			
Gleason					
<7	1.00	Ref.			
7					
Smoking					
Never	1.00	Ref.	1.00	Ref.	
Former	0.82	(0.48–1.39)	0.71	(0.38–1.31)	
Current	0.95	(0.56–1.60)	0.69	(0.38–1.27)	
CCI					
0	1.00	Ref.			*Unadjusted only
1	0.16	(0.07–0.34)	6.62	(0.07–671.22)	
2	0.12	(0.06–0.25)	3.77	(0.02–648.11)	
3+	0.1	(0.05–0.21)	7.12	(0.04–1405.27)	
Erectile dysfunction					
No	1.00	Ref.	1.00	Ref.	
Yes	0.72	(0.39–1.34)	0.7	(0.31–1.57)	

*Note*: Unadjusted and adjusted odds ratios (ORs) with 95% confidence intervals are presented. Multivariable models were restricted to variables with adequate data completeness and stable estimates; variables with sparse categories or extensive missing data (e.g., Gleason score 7) were included in univariable analyses only. Higher ORs indicate greater anxiety.

There were no statistically significant results in the ordinal logistic regression analyses for the FACT‐P EWB cohort (Table 4).

**TABLE 4:** Ordinal logistic regression results for FACT‐P cohort.

	Unadjusted OR	95% CI	Adjusted OR	95% CI	
Age at diagnosis					
<50	1.00	Ref.			*Unadjusted only
50–59	2.92	(0.55–15.35)			
60–69	4.59	(0.89–23.73)			
70–79	4.56	(0.87–23.88)			
BMI					
≤18.5					
18.5–≤25	1.00	Ref.	1.00	Ref.	
25–≤30	1.07	(0.73–1.58)	1.11	(0.67–1.84)	
>30	1.68	(0.88–3.21)	1.63	(0.67–3.98)	
Marital status					
No	1.00	Ref.			*Unadjusted only
Yes	0.85	(0.50–1.46)			
Education					
Less than high school	1.36	(0.89–2.08)			*Unadjusted only
High school	1.00	Ref.			
University	1.47	(0.95–2.28)			
Family history of PCa					
No	1.00	Ref.			*Unadjusted only
Yes	0.90	(0.58–1.41)			
PSA					
<1	1.00	Ref.	1.00	Ref.	
1–4	0.62	(0.18–2.09)	0.22	(0.02–2.19)	
5–9	0.59	(0.18–1.98)	0.25	(0.03–2.48)	
10+	0.74	(0.16–3.48)	0.12	(0.00–5.04)	
T stage					
T1	1.00	Ref.	1.00	Ref.	
T2	0.85	(0.44–1.61)	0.57	(0.21–1.49)	
No. of biopsy cores with cancer					
1–4	1.00	Ref.			
5–9	0.84	(0.27–2.63)	1.12	(0.26–4.85)	
≥10	1.04	(0.06–18.49)	1.29	(0.07–23.29)	
Gleason					
<7	1.00	Ref.			*Unadjusted only
7	1.04	(0.06–18.47)			
Smoking					
Current	1.00	Ref.	1.00	Ref.	
Former	0.83	(0.53–1.31)	0.97	(0.54–1.72)	
Never	1.21	(0.78–1.90)	1.35	(0.77–2.35)	
CCI					
0	1.00	Ref.			*Unadjusted only
1	3.00	(0.29–31.17)			
2	4.93	(0.48–50.32)			
3+	4.20	(0.41–43.17)			
Erectile dysfunction					
No	1.00	Ref.	1.00	Ref.	
Yes	0.91	(0.51–1.62)	0.72	(0.34–1.52)	

*Note*: Unadjusted and adjusted odds ratios (ORs) with 95% confidence intervals are presented. Multivariable models were restricted to variables with adequate data completeness and stable estimates; variables with sparse categories or extensive missing data (e.g., Gleason score 7) were included in univariable analyses only. Higher ORs indicate greater anxiety.

## 4 DISCUSSION

Psychological wellbeing among men on active surveillance for PCa varied across cohorts and was associated with several demographic and clinical factors in the chi squared analyses including obesity, family history of PCa, PSA, cancer stage and comorbidities. However, these associations did not persist after adjusting for relevant confounders in multivariable models, with the exception of family history in the MAX‐PC cohort. This suggests that while certain baseline characteristics may appear influential in isolation, they are not independently predictive of mental wellbeing when considered alongside other variables.

In an editorial, Klotz discusses how the recognised hypothetical risk of AS may distress patients who are prone to anxiety.^14^ Naturally, it is likely there is always going to be a proportion of men who experience distress and/or anxiety when going through AS, while most patients report positive mental wellbeing. Furthermore, it is also possible that patients on AS may choose to switch to another treatment after a few months due to the mental burden AS. Whatever the reason, evidence from systematic reviews is generally positive with regards to mental wellbeing and AS. For example, one systematic review, including results from 34 studies, concluded that there was no adverse impact on psychosocial wellbeing associated with AS, and no differences in wellbeing compared to active treatments.^15^ Similarly, the systematic review by Bellardita et al. found that men on AS did not suffer major negative psychological impacts but stated that longer follow‐up was required.^9^ One further systematic review by Thompson et al. investigated overall quality of life of men on AS up to 5 years post‐treatment. The authors concluded that men on AS reported good long‐term quality of life comparable to men without PCa.^14^ Overall wellbeing was generally favourable; however, a family history of prostate cancer was the only factor independently associated with higher disease‐specific anxiety. This likely reflects increased risk perception or prior adverse cancer experiences. The lack of association with broader emotional wellbeing suggests a targeted effect on cancer‐related anxiety, identifying a subgroup that may benefit from additional support during active surveillance.

Statistically significant associations were only observed with the MAX‐PC questionnaire, whereas the FACT‐P EWB showed no significant variation across baseline characteristics. The contrasting findings likely reflect differences in questionnaire focus. MAX‐PC is a disease‐specific questionnaire meaning it is likely more sensitive to variation in clinical and demographic factors such as BMI, PSA and family history. FACT‐P EWB, however, captures broader emotional wellbeing, which may be shaped by psychosocial influences not measured here, and may also show less variability, limiting detection of associations. These findings highlight the value of incorporating both disease‐specific and general instruments to capture complementary dimensions of mental health. Finally, we would like to note that the observed change in the direction and significance of the association between obesity and anxiety after adjustment, together with the wide confidence interval, suggests that this finding should be interpreted cautiously and may reflect residual confounding, collinearity with other baseline factors or limited power within this subgroup.

Several limitations should be acknowledged. The lack of standardised survey collection across GAP3 centres restricted inclusion to only three centres, reducing power and limiting subgroup analyses (e.g., by ethnicity) as well as generalisability. The use of tertiles for FACT‐P emotional well‐being, in the absence of validated cut‐points, resulted in narrow score ranges and may have limited sensitivity to detect associations.

The use of cross‐sectional baseline data also precludes assessment of changes in wellbeing over time. In addition, information on socio‐economic status would have been useful to include in the analyses. Future research should include longitudinal designs, standardised patient‐reported outcomes and broader psychosocial measures such as social support, personalities, coping style or financial stress, which may better explain variation in general wellbeing. Implementing core outcome sets (COS) across centres could also enhance data consistency. As the name suggests, COS define a minimum set of outcomes that should be measured and reported in clinical studies as well as part of real world data collection from daily clinical practice.^16^ The PIONEER initiative, a European network of excellence for big data in PCa, has developed specific COS for PCa.^16^, ^17^ To ensure a standardised approach to data collection, it is recommended that future clinical studies adopt these COS. Doing so will facilitate the creation of larger, more comprehensive datasets, ultimately driving significant advancements in PCa research and clinical care.

Despite these limitations, the inclusion of diverse baseline characteristics is a strength of the study, enabling a more nuanced exploration of factors associated with psychological wellbeing in men on AS. Furthermore, despite data only being available from three centres, data from over 900 patients was included in the analysis. In addition, these centres were based in Europe and in the United States and therefore encompassed a breadth of PCa patients. Nevertheless, future studies would certainly benefit from including more centres. A further advantage to the study was utilisation of data from two different questionnaires, as it meant the study was not just focusing on PCa‐related anxiety but additionally considering overall emotional wellbeing.

In terms of future directions, subsequent studies could explore whether baseline anxiety levels serve as a reliable predictor of active surveillance (AS) outcomes in patients. For instance, it would be valuable to investigate whether individuals with higher initial anxiety are more likely to opt for definitive treatment—such as surgery or radiation—despite the absence of disease progression or grade reclassification. This could help to explore the role that psychological factors, rather than clinical indicators alone, play in decision‐making during AS.

## 5 CONCLUSION

In summary, this study demonstrates that while certain baseline demographic and clinical characteristics, particularly family history of prostate cancer, are associated with disease‐specific anxiety in men on active surveillance, they do not consistently predict broader emotional wellbeing. Together, these findings underscore the need for routine psychosocial assessment within active surveillance pathways, alongside further longitudinal and multi‐centre studies to better characterise predictors of distress and to inform tailored supportive interventions.

## AUTHOR CONTRIBUTIONS

B. Russell and D. Nieboer performed the statistical analyses. A. Francis, C.P. Pavlovich, M. Peacock, P. Dasgupta, L.D.F. Venderbos, and M. Van Hemelrijck contributed to data interpretation. P. Dasgupta and M. Van Hemelrijck provided overall study oversight and project administration. M. Tazi and B. Russell drafted the manuscript. All authors reviewed the manuscript, contributed important intellectual content, and approved the final version for publication on behalf of the Global Active Surveillance Data Research Platform (GAP3) consortium.

## CONFLICT OF INTEREST STATEMENT

PDG was supported by the Engineering and Physical Sciences Research Council (EPSRC) (grant number EP/Y009800/1), through funding from Responsible AI UK (RAI UK). He also acknowledges funding from the Trustworthy Autonomous Systems (TAS) Hub and UK Research and Innovation (UKRI). He recognises support from the Wellcome Trust for Surgical and Interventional Engineering, the London Institute for Healthcare Engineering (LIHE), the Hinduja‐King's Academy, Alberto Recordati, the King's‐Vattikuti Institute and The Urology Foundation and King's College London (KCL). The remaining authors have no conflicts of interest to declare.

## Data Availability

The data that support the findings of this study are available from the corresponding author upon reasonable request.

## Appendix A Members of The Global Active surveillance data research Platform (GAP3) consortium

GAP3 partners that contributed financially (during 2022–2023 and/or 2025–2026): Christian Pavlovich (Johns Hopkins University, The James Buchanan Brady Urological Institute, Baltimore, USA), Peter Carroll (University of California San Francisco, San Francisco, USA), Christopher Filson (Emory University School of Medicine, Winship Cancer Institute, Atlanta, USA), Michael Peacock (University of British Columbia, BC Cancer Agency, Vancouver, Canada), Eric Hyndman (University of Calgary, Southern Alberta Institute of Urology, Calgary, Canada), Caroline Moore (University College London & University College London Hospital Trust, London, UK), Mieke Van Hemelrijck (King's College London, London, UK & Guy's and St Thomas' NHS Foundation Trust, London, UK), Prokar Dasgupta (Guy's and St Thomas' NHS Foundation Trust, London, UK), Chris Bangma/Monique Roobol (Erasmus Medical Center, Rotterdam, The Netherlands), Philipp Papavassilis/Axel Semjonov (University Hospital Muenster, Muenster, Germany), Antti Rannikko (Helsinki University and Helsinki University Hospital, Helsinki, Finland), Cristina Marenghi/Ricardo Valdagni (Fondazione IRCCS Istituto Nazionale dei Tumori, Milan, Italy), Anders Bjartell (Skåne University Hospital, Malmö, Sweden), Lee Lui Shiong (Singapore General Hospital, Singapore, Singapore), Lui Shiong Lee (Singapore General Hospital, Singapore, Singapore), Mark Frydenberg (Monash University; Cabrini Institute, Cabrini Health, Melbourne, Australia), Mikio Sugimoto (Kagawa University Faculty of Medicine, Kagawa, Japan), Byung Ha Chung (Gangnam Severance Hospital, Yonsei University Health System, Seoul, Republic of Korea).

Principle Investigators: Christian Pavlovich (Johns Hopkins University, The James Buchanan Brady Urological Institute, Baltimore, USA), Behfar Ehdaie (Memorial Sloan Kettering Cancer Center, New York, USA), Peter Carroll (University of California San Francisco, San Francisco, USA), Martin Sanda (Emory University School of Medicine, Winship Cancer Institute, Atlanta, USA), Christopher Logothetis (MD Anderson Cancer Centre, Houston, USA), Kevin Ginsburg (Wayne State University and the Michigan Urological Surgery Improvement Collaborative [MUSIC], Michigan, USA), Laurence Klotz (University of Toronto, Sunnybrook Health Sciences Centre, Toronto, Ontario, Canada), Michael Peacock (University of British Columbia, BC Cancer Agency, Vancouver, Canada), Eric Hyndman (University of Calgary, Southern Alberta Institute of Urology, Calgary, Canada), Caroline Moore (University College London & University College London Hospital Trust, London, UK), Vincent Gnanapragasam (University of Cambridge & Cambridge University Hospitals NHS Foundation Trust, Cambridge, UK), Mieke Van Hemelrijck (King's College London, London, UK & Guy's and St Thomas' NHS Foundation Trust, London, UK), Prokar Dasgupta (Guy's and St Thomas' NHS Foundation Trust, London, UK), Chris Bangma (Erasmus Medical Center, Rotterdam, The Netherlands/ representative of Prostate cancer Research International Active Surveillance [PRIAS] consortium), Monique Roobol (Erasmus Medical Center, Rotterdam, The Netherlands/representative of Prostate cancer Research International Active Surveillance [PRIAS] consortium), The PRIAS study group, Philipp Papavassilis (University Hospital Muenster, Muenster, Germany), Antti Rannikko (Helsinki University and Helsinki University Hospital, Helsinki, Finland), Cristina Marenghi (Fondazione IRCCS Istituto Nazionale dei Tumori, Milan, Italy), Antoinette Perry (University College Dublin, Dublin, Ireland), Augusto Wong Gutierrez (Instituto Valenciano de Oncología, Valencia, Spain), Anders Bjartell (Skåne University Hospital, Malmö, Sweden), Lukas Hefermehl (Kantonsspital Baden, Baden, Switzerland), Lui Shiong Lee (Singapore General Hospital, Singapore, Singapore), Mark Frydenberg (Monash University; Cabrini Institute, Cabrini Health, Melbourne, Australia), Phillip Stricker (St Vincents Prostate Cancer Centre, Sydney, Australia), Mikio Sugimoto (Kagawa University Faculty of Medicine, Kagawa, Japan), Kyochul Koo (Gangnam Severance Hospital, Yonsei University Health System, Seoul, Republic of Korea).

Pathologist: Theo van der Kwast (Princess Margaret Cancer Centre, Toronto, Canada).

Statistical team: Ewout Steyerberg (Erasmus Medical Center, Rotterdam, The Netherlands), Daan Nieboer (Erasmus Medical Center, Rotterdam, The Netherlands); Beth Russell (King's College London, London, UK & Guy's and St Thomas' NHS Foundation Trust, London, UK), Brian Denton (University of Michigan, Michigan, USA), Paul C. Boutros (University of California, Los Angeles, USA).

Clinical Research Partners' IT Experts: Mufaddal Mamawala (Johns Hopkins University, The James Buchanan Brady Urological Institute, Baltimore, USA), Nicole Benfante (Memorial Sloan Kettering Cancer Center, New York, USA), Janet Cowan (University of California San Francisco, San Francisco, USA), Dattatraya Patil (Emory University School of Medicine, Winship Cancer Institute, Atlanta, USA), Lauren Park (MD Anderson Cancer Centre, Houston, Texas, USA), Corinne Labardee/Sabir Meah/(University of Michigan and Michigan Urological Surgery Improvement Collaborative, Ann Arbor, Michigan, USA), Alexandre Mamedov (University of Toronto, Sunnybrook Health Sciences Centre, Toronto, Ontario, Canada), Vincent LaPointe (University of British Columbia, BC Cancer Agency, Vancouver, Canada), Chel Hee Lee (University of Calgary, Southern Alberta Institute of Urology, Calgary, Canada), Cameron Englman (University College London & University College London Hospital Trust, London, UK), Alexandra Keates (University of Cambridge & Cambridge University Hospitals NHS Foundation Trust, Cambridge, UK), Aida Santaolalla (King's College London, London, UK & Guy's and St Thomas' NHS Foundation Trust, London, UK), Daan Nieboer (Erasmus Medical Center, Rotterdam, The Netherlands), Fabio Badenchini (Fondazione IRCCS Istituto Nazionale dei Tumori di Milano, Milan, Italy), Marc Walsh (University College Dublin, Dublin, Ireland), Juanma Mascarós (Instituto Valenciano de Oncología, Valencia, Spain), Annica Löfgren (Skåne University Hospital, Malmö, Sweden), Catherine Han Lin (Monash University and Epworth HealthCare, Melbourne, Australia), Shikha Agrawal (St Vincents Prostate Cancer Centre, Sydney, Australia), Takuma Kato (Kagawa University, Kagawa, Japan), Kwang Suk Lee (Yonsei University College of Medicine, Gangnam Severance Hospital, Seoul, Korea).

Research Advisory Committee: Guido Jenster (Erasmus MC, Rotterdam, the Netherlands), Anssi Auvinen (University of Tampere, Tampere, Finland), Anders Bjartell (Skåne University Hospital, Malmö, Sweden), Masoom Haider (University of Toronto, Toronto, Canada).

Steering committee: Kyochul Koo (Gangnam Severance Hospital, Yonsei University Health System, Seoul, Republic of Korea), Christian Pavlovich (Johns Hopkins University, The James Buchanan Brady Urological Institute, Baltimore, USA), Peter Carroll (University of California San Francisco, San Francisco, USA), Ewout Steyerberg (Erasmus Medical Center, Rotterdam, The Netherlands), Chris Bangma (Erasmus Medical Center, Rotterdam, The Netherlands), Monique Roobol (Erasmus Medical Center, Rotterdam, The Netherlands).

Management team: Jozien Helleman (Erasmus Medical Center, Rotterdam, The Netherlands), Monique Roobol (Erasmus Medical Center, Rotterdam, The Netherlands), Chris Bangma (Erasmus Medical Center, Rotterdam, The Netherlands).
